# Unraveling Signaling Pathways in Immune Microenvironment Crosstalk to Overcome Immunotherapy Resistance in Colorectal Cancer

**DOI:** 10.1155/humu/2744471

**Published:** 2026-02-13

**Authors:** Hui Zhang, Jingjing Shao, Tianye Zhao, Yaxuan Wang, Lili Shao, Haixia Zhu, Jibin Liu

**Affiliations:** ^1^ Department of Clinical Laboratory Medicine, Affiliated Tumor Hospital of Nantong University and Nantong Tumor Hospital, Nantong, Jiangsu, China; ^2^ Cancer Research Center Nantong, Affiliated Tumor Hospital of Nantong University and Nantong Tumor Hospital, Nantong, Jiangsu, China; ^3^ Department of Oncology, Affiliated Tumor Hospital of Nantong University and Nantong Tumor Hospital, Nantong, Jiangsu, China; ^4^ Institute of Oncology, Affiliated Tumor Hospital of Nantong University and Nantong Tumor Hospital, Nantong, Jiangsu, China, onko-i.si

**Keywords:** colorectal cancer, combination therapy, immune evasion, immunotherapy resistance, signaling pathways, tumor microenvironment

## Abstract

As a major cause of cancer‐related death globally, colorectal cancer (CRC) remains largely refractory to immunotherapy outside the context of microsatellite instability‐high (MSI‐H). This limited efficacy stems largely from the complex crosstalk within the tumor microenvironment (TME), which fosters immunosuppression and resistance. Our review analyzes the impact of dysregulated pathways—such as PD‐1/PD‐L1, cGAS/STING, Notch, and cytokine signaling—on the functional states of T cells, B cells, macrophages, dendritic cells, and NK cells in CRC. We investigate how these pathways underpin critical processes such as immune evasion, T cell exhaustion, and the protumor polarization of innate immune cells, thereby fostering a permissive niche for tumor growth and resistance to checkpoint inhibitors. The discussion also covers emerging biomarkers and innovative strategies, including combination therapies targeting pivotal signaling nodes, to reprogram the immune landscape. A deeper mechanistic understanding of these immunoregulatory pathways is essential for developing effective treatments to overcome resistance and improve patient prognosis.

## 1. Introduction

Colorectal cancer (CRC) continues to be a leading contributor to global cancer mortality [1]. It is the third most frequently diagnosed malignancy, responsible for about 7% of new cancer cases and nearly 11% of cancer‐related deaths each year [2, 3]. In China, the incidence and mortality of CRC have seen a significant increase over recent decades, now surpassing global averages and representing a major public health issue [4]. Epidemiological trends suggest the CRC burden in China will continue to grow, underscoring a critical need for improved prevention and treatment. Despite advances in therapeutic options, including chemotherapy, targeted therapy, immune checkpoint inhibitors, and combination regimens, managing CRC remains challenging [5]. Key obstacles include the disease′s complex epigenetic alterations and considerable tumor heterogeneity, which lead to unpredictable treatment responses and the development of resistance [6]. While immunotherapy has revolutionized the treatment of many solid tumors, its efficacy in CRC is largely confined to patients with microsatellite instability‐high (MSI‐H) or mismatch repair‐deficient (dMMR) tumors. In contrast, patients outside this subgroup derive limited benefit and frequently develop resistance. In addition to genetic factors, the tumor microenvironment (TME) plays a critical role in shaping CRC progression and therapeutic outcomes [7–9]. Specifically, the tumor immune microenvironment (TIME) is crucial in modulating cancer behavior and immune evasion [10–12]. The TIME encompasses various immune cells—T lymphocytes, macrophages, neutrophils, myeloid‐derived suppressor cells (MDSCs), and dendritic cells (DCs)—as well as their secreted factors like cytokines and chemokines. These components can directly or indirectly suppress immune responses, promote immune escape, and drive tumor growth and metastasis [13–15]. Hence, a meticulous dissection of the TIME′s constituent elements, functional states, and interplay is indispensable for elucidating the pathophysiological mechanisms driving CRC. Such foundational understanding is equally critical for innovating more potent immunotherapeutic strategies and circumventing resistance, ultimately aiming to ameliorate patient prognoses.

## 2. The Composition of the Immune Microenvironment in CRC

**Figure 1:**
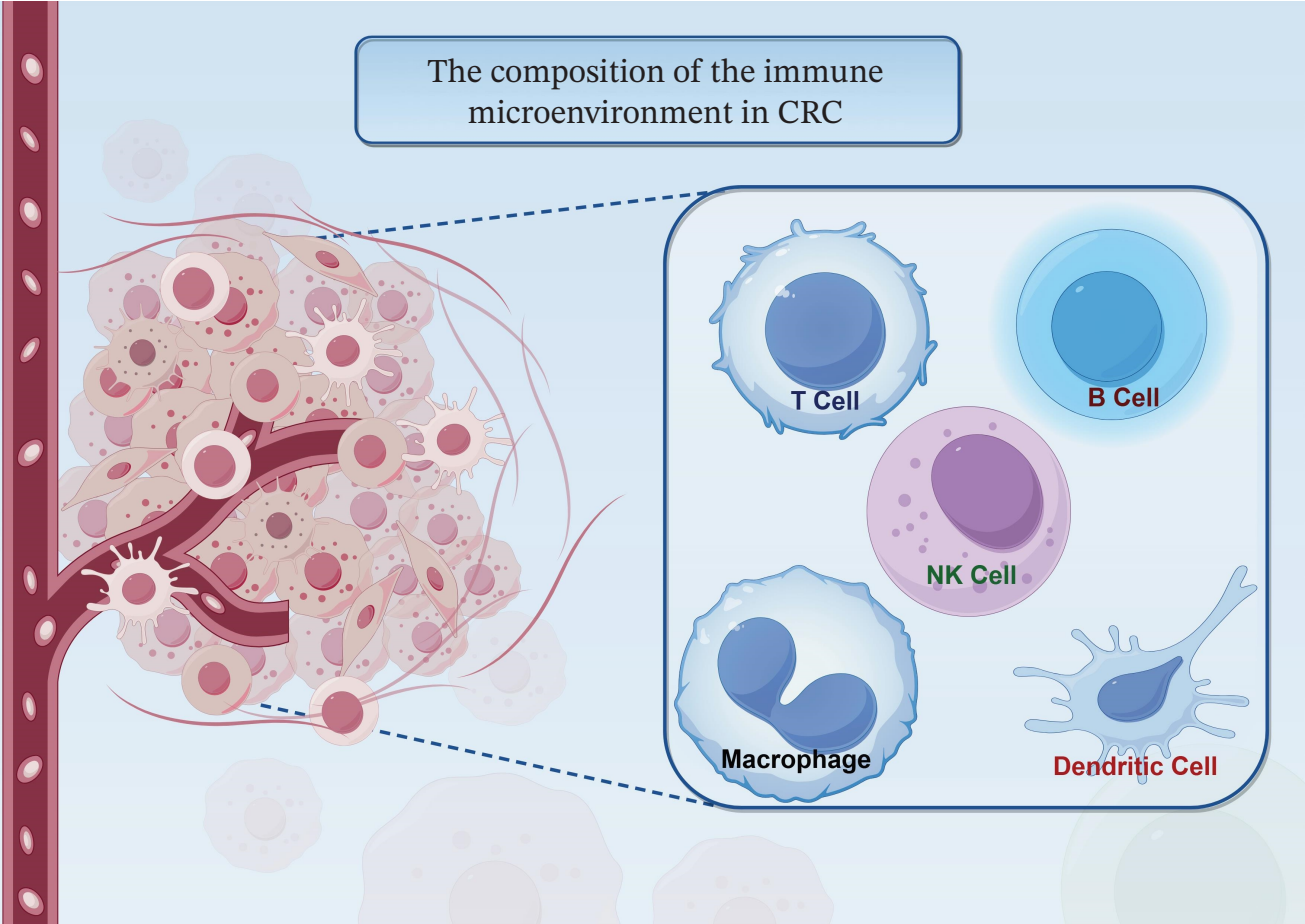
The composition of the immune microenvironment in CRC. The immune microenvironment of colorectal cancer encompasses not only tumor cells but also a variety of immune cells (T cells, B cells, NK cells, macrophages, and dendritic cells), fibroblasts, endothelial cells, and stromal components. The interactions among these elements determine the critical balance between immune homeostasis and pathological dysregulation.

CRC progression is governed by its immune microenvironment, a sophisticated ecosystem comprising a multitude of cellular constituents and molecular signals essential to carcinogenesis, metastatic dissemination, and therapeutic efficacy [16, 17]. This microenvironment encompasses not only tumor cells but also a diverse array of immune cells (T cells, B cells, NK cells, macrophages, and DCs), fibroblasts, endothelial cells, and stromal components. The crosstalk among these elements determines the critical balance between immune homeostasis and pathological dysregulation (Figure 1). Clinical studies have established a correlation between the density and activation status of T and B lymphocytes in CRC tissues and the stage of disease progression [18]. Application of the ESTIMATE algorithm to transcriptomic data from 415 patients yielded a prognostic signature based on immune‐related genes (IRGs), including AXIN2 and CCL22. This analysis further identified a pronounced enrichment of resting NK cells and regulatory T cells (Tregs), coupled with elevated PD‐1 and PD‐L1 levels, in the high‐risk patient subgroup, underscoring the prognostic significance of specific immune contexts [16]. Beyond cellular players, molecular signals are fundamental in configuring the immune landscape. Immune cell dynamics in CRC are influenced by key regulators like the chemokine CCL22, which recruits immunosuppressive Tregs via CCR4 to foster an immune‐evasive environment. Additionally, remodeling of the extracellular matrix actively suppresses immunity beyond providing mere physical obstruction. Thus, the CRC immune microenvironment functions as a cohesive regulatory network, not a collection of isolated elements. Advancing the mechanistic understanding of this network is vital for designing next‐generation immunotherapies.

## 3. Classification and Function of Immune Cells in CRC

CRC involves a diverse array of immune cells that perform specialized functions, collectively shaping disease initiation and progression (Table 1).

**Table 1:** Major immune cell subsets in the colorectal cancer microenvironment: Phenotypes, functions, and roles in tumor progression.

Immune cell	Major subsets	Functional features	Role in CRC
T cells	CD8^+^ cytotoxic T cells	Cytotoxicity, IFN‐*γ* production, tumor cell killing	Antitumor; associated with better prognosis
	Regulatory T cells (Tregs)	Immunosuppression via IL‐10, TGF‐*β*; express CTLA‐4, PD‐1	Protumor; promote immune evasion and resistance
	*γδ* T cells	Tissue surveillance; dual roles in early vs. late CRC	Context‐dependent: Antitumor in normal/preneoplastic tissue, protumor in established tumors
	CXCL13^+^ T cells	Associated with tertiary lymphoid structures (TLSs)	May support antitumor immunity

B cells	IgG^+^ plasma cells	Antibody production, antigen presentation	Antitumor; correlate with better prognosis
	IgA^+^ plasma cells	Mucosal immunity, dominant in normal mucosa	Homeostatic; may be suppressed in tumors
	Erbin^+^ B cells	Promote metastasis via TGF‐*β*/STAT6 signaling	Protumor; support metastatic niche formation

NK cells	CD56dimCD16^+^	High cytotoxicity, perforin/granzyme‐mediated killing	Antitumor; often functionally impaired in CRC
	CD56bright	Cytokine production (IFN‐*γ*, TNF‐*α*), immunomodulation	Regulatory; may exhibit reduced cytotoxicity

Macrophages	M1‐like TAMs	Proinflammatory, express iNOS, CD86, promote phagocytosis	Antitumor; often suppressed in CRC TME
	M2‐like TAMs	Immunosuppressive, express CD206, Arg‐1, promote angiogenesis	Protumor; correlate with advanced stage and resistance

Dendritic cells	cDC1 (conventional type 1)	Cross‐presentation, CD8^+^ T cell priming	Antitumor; essential for effective immunity
	Immature/tolerogenic DCs	Induce T cell tolerance, express low MHC‐II and costimulators	Protumor; facilitate immune evasion

### 3.1. T Cell

T cells in CRC exhibit context‐dependent roles, either suppressing or promoting tumor growth based on their subtype and signals within the microenvironment. CD8^+^ T lymphocytes, as primary cytotoxic effectors, are linked to improved patient survival when they infiltrate the tumor site [19]. Mismatch repair‐deficient (dMMR) tumors produce neoantigens that—together with costimulatory signals—prompt potent CD8^+^ T cell activation [20]. Recruitment of these cells is further mediated by chemokines such as CCL5 and CXCL10, whose expression is driven by the cGAS/STING pathway and Type I interferon signaling [21]. This chemokine‐guided migration supports the establishment of tissue‐resident memory‐like T cells, which are vital for durable tumor control [22]. Conventional chemotherapy can also induce these chemokines, offering a potential strategy to boost T cell infiltration even in tumors with low neoantigen load [23]. Other T cell populations also contribute to immune regulation. *γδ* T cells exert antitumor activity in precancerous or normal colon tissue but often adopt a protumorigenic state within established CRC lesions [24], a shift associated with altered T cell receptor usage [25]. Additionally, Tregs and exhausted T cells foster immunosuppression via cytokines such as IL‐10 and TGF‐*β* and through direct contact with DCs and macrophages [26]. Tregs also interact with tumor‐associated macrophages (TAMs), further amplifying immune suppression [27]. Single‐cell analyses have revealed distinct T cell subsets in CRC tissues—including CXCL13^+^ T cells and Th17 cells—highlighting the functional diversity within the TME [28]. Crosstalk with other immune cells also modulates T cell activity; for example, tumor‐infiltrating neutrophils can alter CD8^+^ effector memory T cells, inducing a protumor phenotype characterized by high Granzyme K expression [29]. Overall, T cell function in CRC is shaped by a dynamic network of chemokines, cytokines, and cellular interactions.

### 3.2. B Cell

B cells contribute to humoral immunity and display considerable functional plasticity within the CRC microenvironment. Infiltration of B lymphocytes often correlates with favorable prognosis, suggesting a role in antitumor immunity [30]. An enriched IgG^+^ plasma cell compartment in tumors indicates an active, tumor‐evoked B cell response, which may have diagnostic relevance [23, 30]. Class‐switch recombination and somatic hypermutation allow B cells to refine antigen‐specific antibody production, directly influencing immune recognition [30]. However, certain B cell subsets can promote metastasis. For instance, in lung metastatic niches, Erbin‐positive B cells support this process by impairing IgA^+^ cell migration via TGF*β*‐mediated signaling and upregulating PD1 expression through STAT6 activation [31]. Single‐cell RNA sequencing has identified tissue‐resident memory B cells and plasma cells as key subsets, with IgG^+^ plasma cells accumulating in tumor tissue and IgA^+^ plasma cells predominating in adjacent normal mucosa [27, 31]. B cell receptor repertoire analysis reveals selective use of immunoglobulin variable region genes such as IGLV2‐8 and IGLV3‐25 in tumors, implying antigen‐driven selection or microbiota‐related skewing [27]. The gut microbiome further influences B cell function, underscoring its role in CRC immunity [27, 30]. Thus, B cells play multifaceted roles in CRC pathogenesis, spanning immune regulation, microenvironment remodeling, and antibody‐mediated responses.

### 3.3. NK Cell

Natural killer cells provide innate immune surveillance against tumors without prior antigen exposure, making them attractive targets for immunotherapy [32]. In CRC, however, NK cell function is frequently impaired, showing reduced cytotoxicity and cytokine production that correlate with advanced disease and poorer outcomes [33]. Immunophenotyping reveals a decline in the CD56dimCD16^+^ NK subpopulation—which mediates strong tumor killing—alongside an increase in less cytotoxic CD56bright and CD56dimCD16^-^ subsets [34]. Functional defects involve downregulation of perforin, granzyme B, and IFN‐*γ* [35], with suppression becoming more pronounced as tumors progress [36]. NK cells also modulate adaptive immunity by interacting with DCs, enhancing their maturation and subsequent T cell activation [37]. Toll‐like receptor agonists such as R848 can amplify this crosstalk, promoting an inflammatory milieu and improving tumor control [38]. Additionally, NK cells display potent activity against cancer stem cells (CSCs); IL‐2‐activated NK cells effectively target CSCs, suggesting a therapeutic strategy to prevent recurrence [39, 40]. Despite these functions, the immunosuppressive TME often restrains NK cell activity in CRC.

### 3.4. Macrophage

TAMs demonstrate high plasticity in CRC, adopting either antitumor (M1‐like) or protumor (M2‐like) phenotypes depending on microenvironmental cues [41, 42, 43]. M2‐like TAMs, marked by CD206 and Arg‐1, produce IL‐10 and TGF‐*β* to support immune evasion, angiogenesis, and tissue remodeling [41–43]. In contrast, M1‐like TAMs express CD86 and iNOS and promote inflammation and tumor cell phagocytosis [41, 43]. A shift toward the M2 phenotype often correlates with advanced disease and treatment resistance [42, 43]. Tumor‐derived factors and extracellular matrix components drive TAM polarization toward a protumor state, characterized by reduced MHC‐II and elevated CD206 expression [42]. This reprogramming involves miRNA‐mediated suppression of CIITA, a master regulator of MHC‐II, via miR146b and let‐7i [42]. Notch signaling—particularly through Notch3—also recruits immunosuppressive myeloid cells, reinforcing a permissive TME [43]. Conversely, Group 1 innate lymphoid cells (ILC1s) can promote M1‐like polarization through IFN‐*γ*, suggesting avenues for therapeutic intervention [44]. Current strategies are aimed at reprogramming TAMs toward an M1‐like state or disrupting protumor signaling pathways such as Notch [43, 44].

### 3.5. DC

DCs orchestrate antitumor immunity by presenting tumor antigens and priming T cell responses in CRC [45, 46]. Their functional state—ranging from immunogenic to tolerogenic—critically influences outcome. While mature conventional type 1 DCs (cDC1s) excel in cross‐presenting antigens and activating CD8^+^ T cells, immature DCs tend to induce tolerance [45, 47]. CRC often impairs DC maturation, facilitating immune evasion [45]. Effective immunity requires DC migration to tumor‐draining lymph nodes, a process disrupted in advanced disease due to impaired cGMP synthesis. Phosphodiesterase 5 (PDE5) inhibition can restore DC migration and enhance T cell priming [46]. Notch2 signaling is another key regulator of cDC1 function; its disruption compromises DC‐mediated T cell activation and supports tumor development [48]. Immunosuppressive elements such as MDSCs and TAMs further inhibit DC activity [45, 46], while altered expression of IL‐33 and RAB proteins correlates with dysfunctional DC–T cell crosstalk [48]. Therapeutic approaches using DC‐based vaccines or adoptive transfer of Notch‐primed DCs show promise in preclinical models [46, 48]. Combining PDE5 inhibition or Notch modulation with checkpoint blockade may offer new avenues for CRC immunotherapy.

## 4. Immune Cell–Related Biomarkers in CRC

The quest for immune‐based biomarkers in CRC is driven by their significant potential to improve diagnostic accuracy and therapeutic outcomes. Among the most promising candidates are the STAT family proteins, STAT3 and STAT5B, which are frequently downregulated in CRC and linked to favorable survival. Their participation in critical immune and inflammatory processes suggests utility not only as prognostic indicators but also as actionable targets for immunotherapy [49]. Building on this, the prognostic power of tumor‐infiltrating immune cells (TIICs) is exemplified by gene expression models that effectively predict survival and immunotherapy responsiveness, cementing the role of immune signatures in clinical decision‐making [50]. Furthermore, the cytokine IL‐1*β* promotes CRC progression by modulating immune cell infiltration and DNA methylation status, thereby influencing the PI3K/Akt pathway. This central role underscores its dual value as both a promising biomarker and a therapeutic target [51]. Another key player is the exosomal protein S100A11, which acts as a diagnostic biomarker by driving CRC progression through modifications of the immune microenvironment, thereby highlighting exosomes as vessels for biomarker discovery [52]. Mendelian randomization analyses lend causal support by connecting specific immune cell traits to CRC development, positioning them as tools for risk stratification and preventive targeting [53, 54]. The broader immune macroenvironment, characterized by elements like tertiary lymphoid structures (TLSs) and immune checkpoint expression, is equally critical. Positive clinical outcomes are associated with both TLS presence and the effective use of checkpoint inhibitors [55]. Specifically, immune checkpoints such as PD‐1 and PD‐L1 are well‐established biomarkers whose overexpression often predicts advanced disease and inferior survival, confirming their relevance for prognosis and treatment prediction [56]. Adding another layer, transcriptional studies of macrophage subpopulations provide valuable prognostic information, demonstrating how the evolving immune contexture influences responses to immunotherapy [57]. In summary, a broad array of immune‐related biomarkers, ranging from signaling molecules and exosomal cargo to specific cellular and structural components of the TME, holds significant potential for refining clinical strategies in CRC (Table 2). A deeper, mechanistic understanding of this dynamic immune landscape will be fundamental to realizing more precise and effective therapeutic interventions.

**Table 2:** Selected immune‐related biomarkers in colorectal cancer and their clinical relevance.

Biomarker	Type	Expression/status and prognosis	Potential clinical utility
STAT3/STAT5B	Signaling proteins	Downregulation → better survival	Prognostic markers; potential therapeutic targets
PD‐1/PD‐L1	Immune checkpoints	High expression → advanced disease, poorer prognosis	Predictors of response to immune checkpoint inhibitors
cGAS/STING pathway	Innate immune signaling	Activation → enhanced T cell recruitment (via CCL5/CXCL10)	Target for combination immunotherapy
IL‐1*β*	Cytokine	High expression → tumor progression, immune suppression	Prognostic biomarker; therapeutic target
S100A11 (exosomal)	Exosomal protein	Upregulated in CRC patients → diagnostic potential	Liquid biopsy biomarker for early detection
Tertiary lymphoid structures (TLS)	Histological structure	Presence → improved prognosis and immunotherapy response	Predictive marker for immunotherapy benefit
Tumor‐infiltrating lymphocytes (TILs)	Cellular signature	High CD8^+^ T cell density → better chemotherapy response	Prognostic and predictive of treatment outcome
AXIN2, CCL22 (IRG signature)	Immune‐related genes	Enriched in high‐risk subgroup → poor prognosis	Prognostic gene signature for risk stratification

## 5. The Role of Immune Cells in Treatment Resistance in CRC

Treatment resistance remains a major obstacle in CRC, adversely affecting patient survival and quality of life. A growing body of evidence underscores the pivotal contribution of immune cells within the TME to these resistance mechanisms. The dynamic interplay between immune populations and tumor cells not only fuels cancer progression but is also intrinsically linked to therapeutic efficacy and the emergence of resistance (Table 3). The density and spatial organization of TIICs hold significant prognostic value in CRC. Although abundant immune infiltration can sometimes correlate with unfavorable outcomes, the specific composition of this infiltrate is critical; certain immune cell subsets are predictive of a more favorable response to treatment [58, 59]. For example, a high density of CD8^+^ T cells is generally associated with better chemotherapy response, whereas an enrichment of Tregs often fosters an immunosuppressive milieu that promotes immune evasion and resistance [60, 61]. A central mechanism of resistance is immune escape, whereby tumor cells evade destruction by upregulating immune checkpoint molecules like PD‐L1 to inactivate T cells [62, 63]. This process is further aided by immunosuppressive cells in the TME, such as MDSCs and Tregs, which secrete a range of inhibitory factors to dampen antitumor immunity [64, 65]. These mechanisms can confer resistance not only to immunotherapy but also to conventional chemotherapy [66, 67]. The molecular underpinnings of immune‐mediated resistance are complex, involving multiple genes and pathways. Altered expression of genes like *USP20* and *SMAD4* has been linked to both modulation of immune infiltration and chemoresistance [67, 68]. Furthermore, CSCs—with their inherent capacity for self‐renewal and differentiation—represent another key source of resistance, as they can survive initial chemotherapy and lead to disease relapse [69]. In conclusion, immune cells are integral to the development of treatment resistance in CRC. A deeper understanding of their interactions within the TME is essential for devising novel strategies to overcome therapeutic failure and improve clinical outcomes [70, 71]. Elucidating these roles will provide a critical foundation for personalizing CRC therapy [72, 73].

**Table 3:** Mechanisms of immune cell–mediated treatment resistance in colorectal cancer.

Immune cell subset	Key mechanisms of resistance
Regulatory T cells (Tregs)	Secrete IL‐10, TGF‐*β*; suppress CD8^+^ T cell function; interact with TAMs to amplify immunosuppression
M2‐like macrophages	Promote angiogenesis, matrix remodeling, and T cell suppression; express PD‐L1; contribute to checkpoint inhibitor resistance
Exhausted T cells	Upregulate PD‐1, TIM‐3, LAG‐3; lose proliferative and cytotoxic capacity; resist reinvigoration by checkpoint blockade
Myeloid‐derived suppressor cells (MDSCs)	Inhibit T cell activation via arginase, iNOS, ROS; promote Treg expansion
Protumor B cell subsets	Support metastatic niche via TGF‐*β* signaling; upregulate PD‐1 in T cells through STAT6 activation
Cancer stem cells (CSCs)	Evade immune detection through low MHC‐I expression; resist chemotherapy and promote relapse
Dysfunctional dendritic cells	Impaired migration and antigen presentation; fail to prime T cells; express tolerogenic molecules

## 6. Future Perspectives

Advances in deciphering the TIME of CRC are paving the way for innovative research and therapeutic strategies. Several key directions are anticipated to drive progress in immunotherapy and precision oncology for CRC in the coming years. First, resolving the functional heterogeneity and plasticity of immune cells within the TME will require the continued application of high‐resolution technologies, such as single‐cell multiomics and spatial transcriptomics. These tools can map cellular states, lineage dynamics, and communication networks at unprecedented detail, revealing novel immune subsets—such as context‐dependent macrophages, DCs, or T cells—that dictate immune evasion or therapeutic response. To this end, integrating these multidimensional datasets with clinical information is critical for building robust prognostic models and uncovering new therapeutic vulnerabilities. Concurrently, overcoming resistance to existing immunotherapies—especially in poorly responsive microsatellite‐stable (MSS) tumors—represents a priority. This will require a concerted focus on reversing the immunosuppressive nature of the TME. Promising approaches include reprogramming TAMs by targeting polarization signals (e.g., Notch or CSF1R), enhancing DC migration and function (e.g., via PDE5 inhibition or Notch agonism), and reversing T cell exhaustion. Combining ICIs with modulators of chemokine signaling (e.g., CXCR3 or CCR5 antagonists) may also convert immunologically “cold” tumors into “hot” ones by improving T cell recruitment. Finally, the roles of nonclassical immune players, including ILCs, *γδ* T cells, and B cell subsets, warrant deeper investigation. Their dual roles in CRC pathogenesis suggest that future therapies may need context‐specific modulation rather than blanket activation or suppression. Strategies such as developing vaccines to stimulate antitumor B cell responses or selectively inhibiting protumorigenic plasma cells could open new therapeutic avenues. Ultimately, a more nuanced understanding of the entire immune ecosystem will be crucial for developing effective, personalized combination therapies for CRC patients.

## Author Contributions

**Hui Zhang:** writing – review and editing, writing – original draft. **Jingjing Shao:** writing – review and editing, conceptualization, supervision. **Tianye Zhao, Yaxuan Wang:** conceptualization, supervision. **Lili Shao, Haixia Zhu, and Jibin Liu:** supervision, writing – review and editing, funding acquisition, conceptualization. **Hui Zhang and Jingjing Shao:** contributed equally to this work.

## Funding

This study was supported by the Nantong Health Commission Research Project (MS2022053, QN2023025, and QN2024026) and the Nantong Science and Technology Bureau Project (MSZ2024121).

## Conflicts of Interest

The authors declare no conflicts of interest.

## Data Availability

The data that support the findings of this study are available from the corresponding authors upon reasonable request.
